# Case Report: Unrepaired Tetralogy of Fallot in a 50-year-Old woman with 13 pregnancies: multimodal cardiovascular imaging, surgical repair, and longitudinal remodeling

**DOI:** 10.3389/fcvm.2026.1763017

**Published:** 2026-07-07

**Authors:** Moath Hattab, Yahya Ismail, Ahmed Darsalim, Nour Deek, Mohammad Abed, Adham Abderrazeq, Abelhalim Abuhaltam, Mohammed SalahAldin, Mohammed Abutaqa, Nizar Hijjeh

**Affiliations:** 1 Department of Pediatric Cardiology and Cardiothoracic Surgery, Palestinian Medical Complex, Ramallah, Palestine; 2 Department of Medicine, Faculty of Medicine and Allied Medical Sciences, An-Najah National University, Nablus, Palestine; 3 Department of Cardiology, An-Najah National University Hospital, Nablus, Palestine

**Keywords:** adult congenital heart disease, cyanotic heart disease, multimodal cardiovascular imaging, right ventricular outflow tract obstruction, Tetralogy of Fallot

## Abstract

**Background:**

Survival into adulthood without repair of Tetralogy of Fallot (TOF) is exceedingly rare, with only 3%–5% of patients reaching 40 years without intervention. Cardiovascular imaging is central to establishing late diagnoses and planning complex surgical repair.

**Case Summary:**

We report a 50-year-old woman with no prior cardiac diagnosis who presented with progressive dyspnea and profound cyanosis. Despite 13 pregnancies and 11 successful deliveries, she had never been evaluated for congenital heart disease. Multimodal cardiovascular imaging, including transthoracic (TTE) and transesophageal echocardiography (TEE), color Doppler, contrast studies, cardiac catheterization, and chest radiography, demonstrated classic TOF anatomy: a large sub-aortic VSD, overriding aorta, severe right ventricular outflow tract (RVOT) obstruction, hypoplastic pulmonary annulus, and secondary right-sided dilation.

The patient underwent successful corrective surgery with VSD patch closure, pulmonary valve commissurotomy, and pulmonary artery plasty. Postoperatively, oxygen saturation normalized, and serial echocardiography over 6 months showed substantial reduction in RV/RA dimensions and improvement in peak RVOT gradients, although significant residual RVOT obstruction persisted at 6 months. (RV diameter 4.38 → 3.98 cm, RA area 23.4 → 17.9 cm², RVOT gradient 124 → 67 mmHg).

**Conclusion:**

This case highlights the diagnostic value of multimodal cardiovascular imaging in detecting unrecognized cyanotic congenital heart disease in adults and demonstrates that late TOF repair can be safe and effective. It underscores the need for routine interpretation of unexpectedly low oxygen saturation in adults and strengthened awareness among cardiologists, echocardiographers, and general physicians of congenital heart disease beyond childhood.

## Introduction

**Figure 1:**
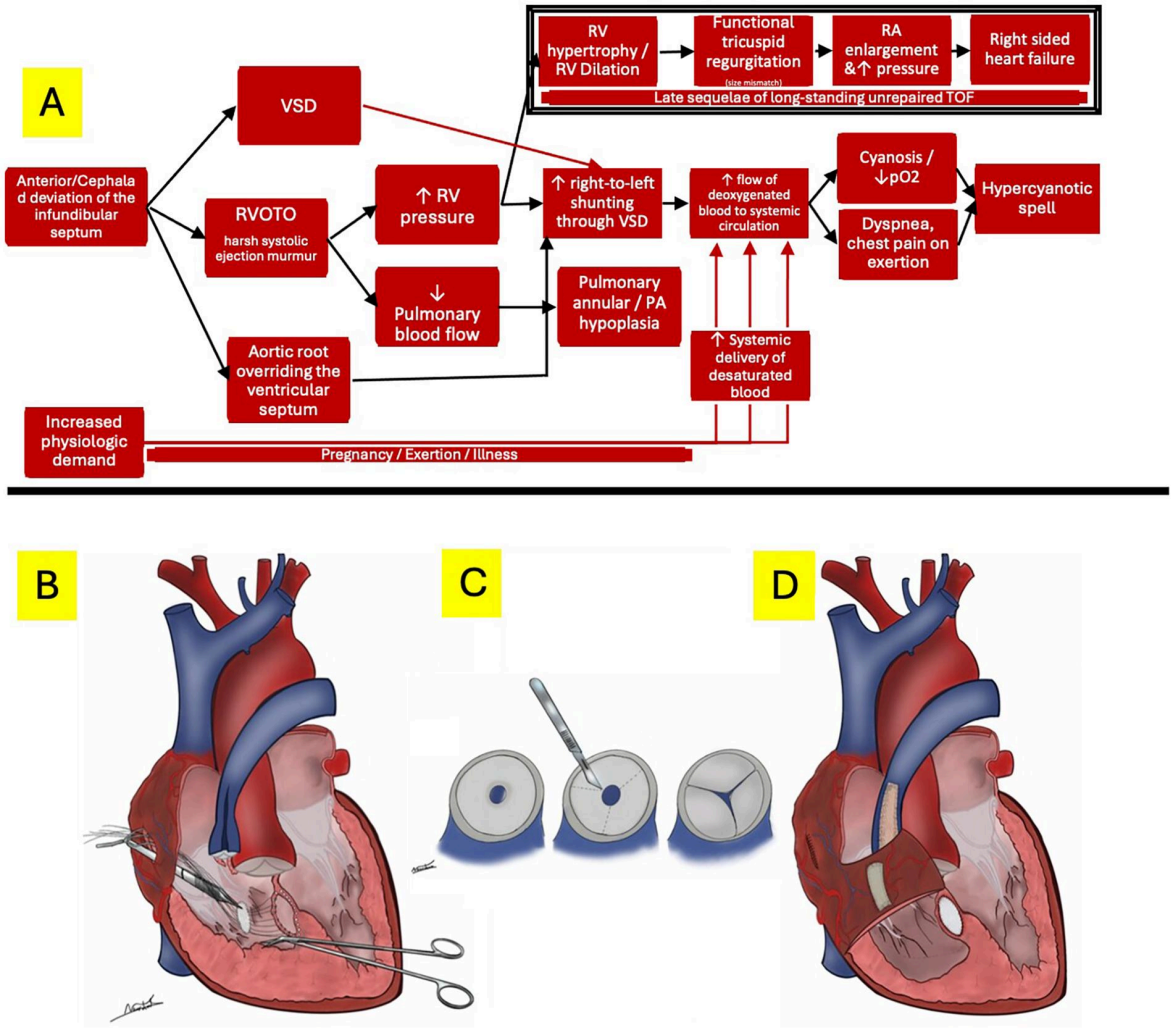
Pathophysiology, chronic remodeling, and case-specific valve-sparing repair in long-standing unrepaired tetralogy of Fallot. **(A)** Schematic summarizing the major anatomic and hemodynamic features relevant to this patient, including anterior-cephalad deviation of the infundibular septum, ventricular septal defect, overriding aorta, right ventricular outflow tract obstruction, reduced pulmonary blood flow, increased right ventricular pressure, right-to-left shunting through the ventricular septal defect, systemic desaturation, cyanosis, and exertional symptoms. The remodeling pathway illustrates late sequelae of long-standing unrepaired TOF, including right ventricular hypertrophy and/or dilation, functional tricuspid regurgitation, right atrial enlargement/increased right atrial pressure, and right-sided heart failure. Pulmonary hypertension is not depicted as a direct sequela because it is not a typical feature of classic unrepaired TOF with severe RVOT obstruction. **(B–D)** Case-specific valve-sparing operative repair performed in this patient. The surgical panels illustrate the individualized operative strategy used in this patient rather than a standard repair for all patients with TOF. **(B)** Transatrial/trans-tricuspid and right ventricular outflow tract exposure. **(C)** Pulmonary valve commissurotomy. **(D)** Completed valve-sparing repair, including ventricular septal defect patch closure, right ventricular outflow tract muscle resection, and pulmonary artery augmentation with treated pericardium.

Tetralogy of Fallot (TOF) is the most common form of cyanotic congenital heart disease and is characterized by four cardinal features: a ventricular septal defect, right ventricular outflow tract obstruction (RVOTO), an overriding aorta, and right ventricular hypertrophy. Although TOF is classically defined by ventricular septal defect, right ventricular outflow tract obstruction, overriding aorta, and right ventricular hypertrophy, these features arise from anterior and cephalad deviation of the infundibular septum during embryologic development. Figure 1 summarizes the pathophysiology and chronic remodeling relevant to this patient. The prevalence is about 3 in every 10,000 live births, making it the most common cause of cyanotic heart disease and about 10% of all congenital cardiac malformations (1). Males and females are affected equally (2). Historically, the natural history of unrepaired TOF has been poor, with survival decreasing sharply after childhood, starting with 75% at the first year, 60% by the third year, 30% at the 10th year, 11% at 20 years, and only an estimated 3%–5% of patients reaching the age of 40 without surgical intervention due to hypoxic spells, cerebrovascular events, and brain abscesses, which are the three most common causes of death in this population (1). Advances in diagnostic imaging and surgical techniques have dramatically improved outcomes, resulting in a growing population of adults with repaired TOF and the emergence of adult congenital heart disease (ACHD) as a dedicated subspecialty (3, 4). In contrast, truly unrecognized and unrepaired TOF in adults is now exceedingly rare, particularly in women who have undergone multiple pregnancies, given the substantial hemodynamic burden imposed by gestation and delivery. Multimodal cardiovascular imaging plays a pivotal role in such late and atypical presentations, not only in establishing the diagnosis, delineating complex anatomy and enabling risk assessment, but also in guiding surgical planning and assessing postoperative longitudinal remodeling. We present an exceptional case of a 50-year-old woman with previously undiagnosed TOF, in whom multimodal cardiovascular imaging was central to diagnosis, operative strategy, and follow-up, and who tolerated a valve-sparing repair with marked clinical improvement and echocardiographic findings suggestive of favorable right-sided remodeling, despite persistent residual RVOT obstruction.

## Case presentation

**Figure 2:**
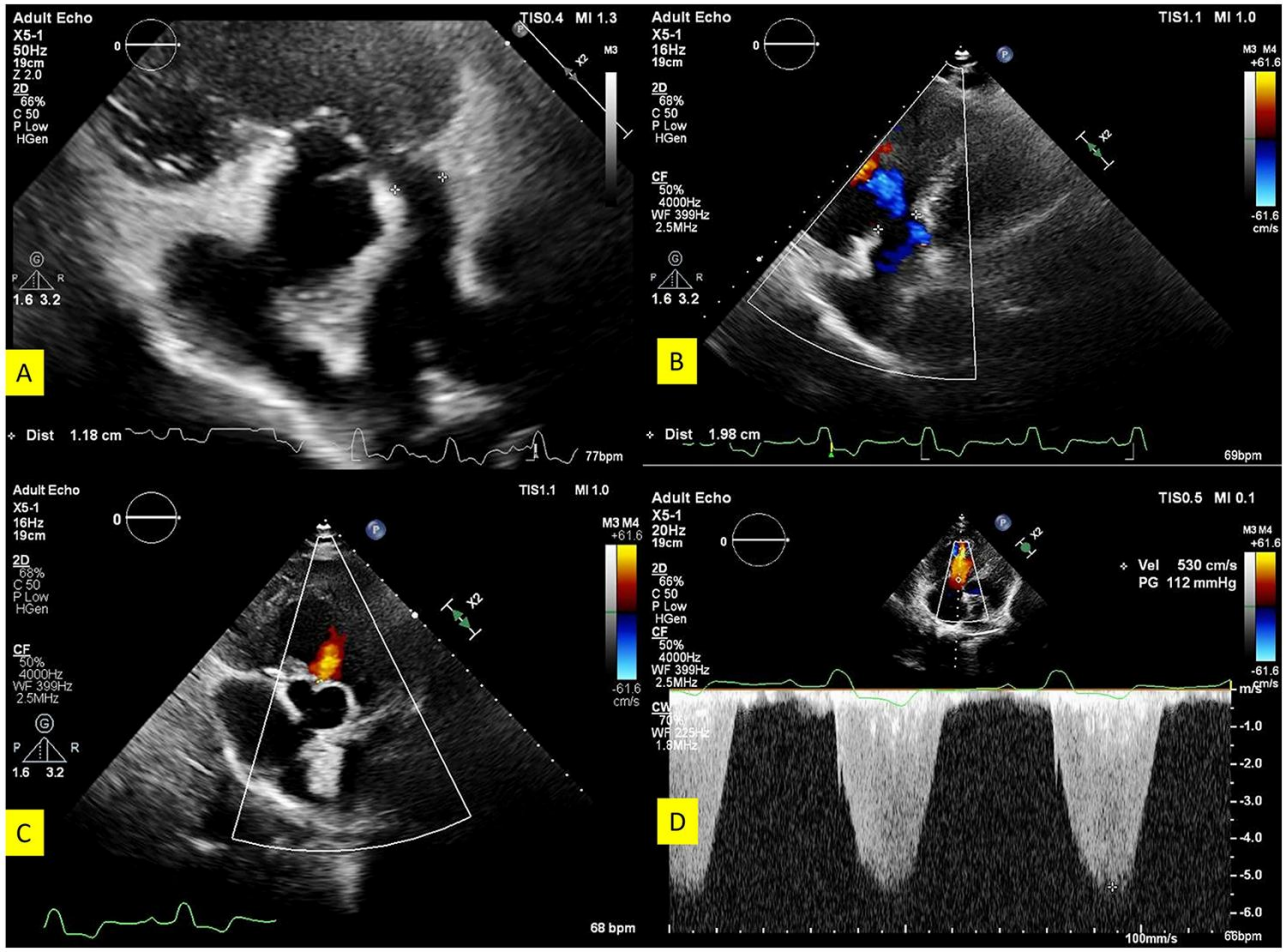
Pre-operative echocardiography diagnosing TOF, **(A)** main pulmonary artery hypoplasia (1.18 cm), **(B)** a 2 cm VSD, **(C)** pulmonic stenosis, **(D)** tricuspid regurgitation.

**Figure 3:**
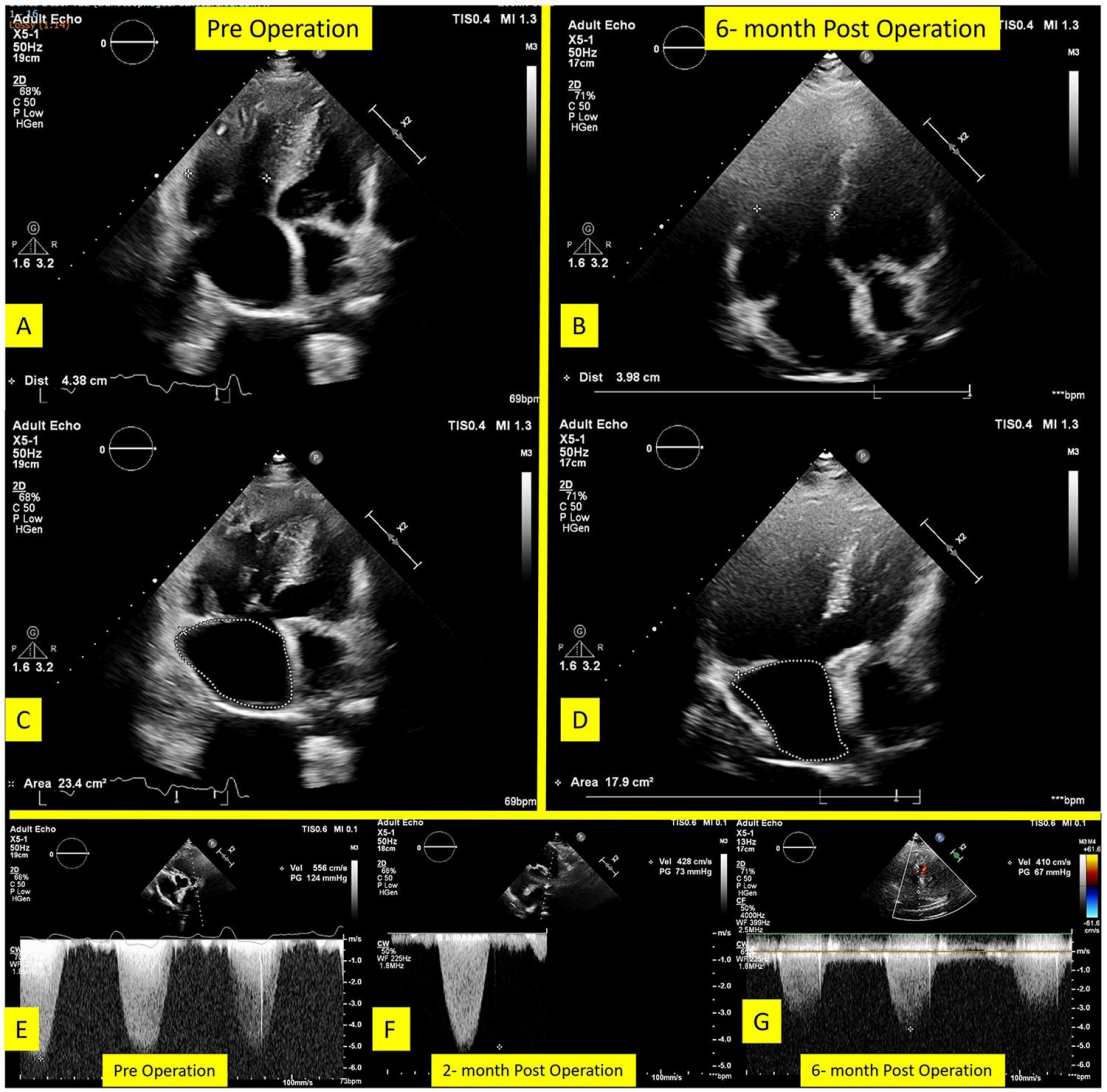
Serial echocardiographic findings before and after surgical repair. Panels **(A)** and **(B)** show a reduction in right ventricular diameter from 4.38 cm preoperatively to 3.98 cm at six months. Panels **(C)** and **(D)** show a reduction in right atrial area from 23.4 cm² preoperatively to 17.9 cm² at six months. Panels **(E–G)** show reduction in the RVOT/pulmonary valve gradient from 124 mmHg preoperatively to 73 mmHg at two months and 67 mmHg at six months. These echocardiographic changes suggest postoperative improvement but do not replace CMR-based quantification of right ventricular volumes and function. The residual RVOT gradient remained significant and requires ongoing ACHD follow-up.

**Figure 4:**
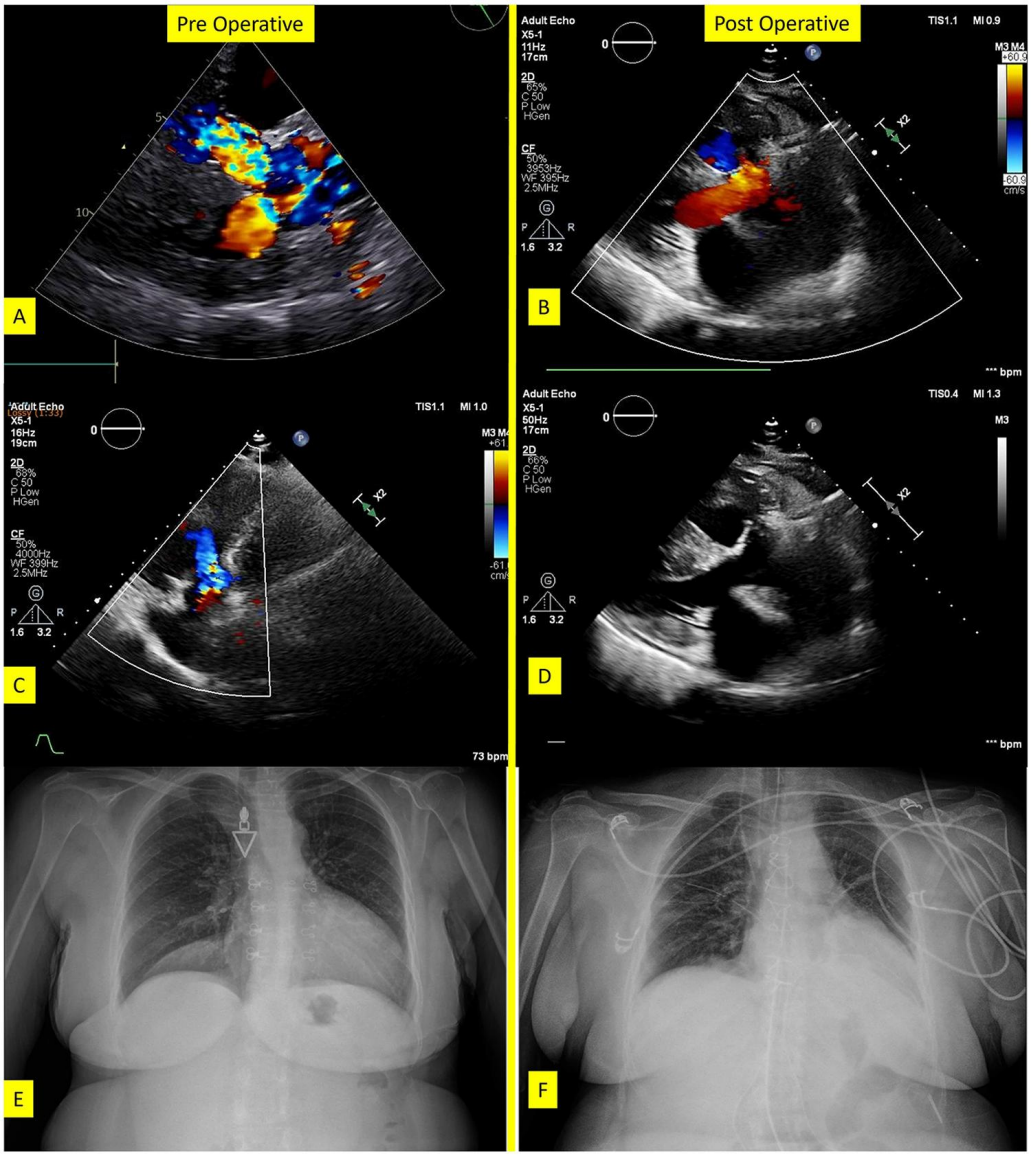
**(A,C)** Pre-operative VSD showing mixing. **(B,D)** Postoperative closed VSD with a patch and no residual mixing. **(E)** pre-operative chest x-ray (CXR) showing boot-shaped heart; **(F)** post-operative CXR. The linear radiopaque densities projected over the sternum in section E are external clothing-related artifacts and do not represent sternal wires. The patient had no history of prior median sternotomy or cardiac surgery.

A 50-year-old woman presented with a one-month history of progressive exertional dyspnea and chest discomfort that had recently progressed from occurring only with exertion to occurring at rest, culminating in an acute episode of severe whole-body cyanosis. She had a history of 13 pregnancies with 11 live births and two miscarriages, with no prior diagnosis of cardiac disease despite routine antenatal care and hospital deliveries. Based on the available history, her pregnancies were not complicated by documented maternal cardiac decompensation, cyanotic spells requiring admission, stroke, thromboembolism, or infective endocarditis. The available obstetric history did not identify offspring with known congenital heart disease. However, detailed obstetric and neonatal records were incomplete, so information regarding gestational age at delivery, mode of delivery, neonatal birthweight, and neonatal intensive care admission could not be reliably reconstructed. She reported smoking approximately two packs of cigarettes per day since the age of 14. On examination, she was centrally and peripherally cyanotic with an oxygen saturation of 65%, while her blood pressure, heart rate, and temperature were within normal limits. On physical examination, cardiac auscultation revealed a systolic murmur best heard along the left upper sternal border. ECG showed sinus rhythm with right bundle branch block and right-sided strain. Laboratory studies demonstrated elevated hemoglobin, possibly attributed to secondary erythrocytosis from chronic hypoxemia. Transthoracic and transesophageal echocardiography were performed and unexpectedly revealed Tetralogy of Fallot, with a large sub-aortic ventricular septal defect of approximately 2 cm with right-to-left shunting, an overriding aorta, severe RVOT obstruction with a hypoplastic pulmonary valve annulus measuring 1.1–1.3 cm, and right ventricular and right atrial enlargement, with the right ventricular diameter measuring 4.38 cm and the right atrial area 23.4 cm², although global left and right ventricular systolic function was preserved (Figures 2, 3). Color Doppler demonstrated a high-velocity jet across the RVOT with a peak gradient of 124 mmHg. Contrast-enhanced TEE raised suspicion for a patent foramen ovale; however, the available still image was not considered sufficiently definitive for publication. Chest x-ray showed the classic boot-shaped cardiac silhouette of TOF (Figure 4).

Cardiac catheterization confirmed severe RVOT obstruction, with a peak-to-peak gradient of 99 mmHg and recorded pressures of 136/11/45 mmHg in the right ventricle and 37/15/25 mmHg in the pulmonary artery, corresponding to a mildly elevated mean pulmonary artery pressure of 25 mmHg. Coronary angiography revealed only mild non-obstructive coronary artery disease. Pulmonary vascular resistance, pulmonary capillary wedge pressure, and Qp:Qs were not available from the catheterization report, limiting definitive assessment of pulmonary vascular disease. Cardiac magnetic resonance imaging was not clinically available in the country at the time of this case and was therefore not performed; our center has since become the first in the country to introduce this service. Cardiac CT was also not performed, as the treating team considered the combination of TTE, TEE, contrast echocardiography, invasive catheterization, coronary angiography, and intraoperative assessment sufficient for diagnosis and operative planning. Based on the available imaging, catheterization, and surgical assessment, the patient was deemed suitable for corrective surgery with a valve-sparing strategy.

Through a median sternotomy under cardiopulmonary bypass and cardioplegic arrest, a right atriotomy with a trans-tricuspid approach and a right ventriculotomy provided exposure; the VSD was closed with a Dacron patch, abolishing the right-to-left shunt, muscular bundles contributing to RVOT obstruction were resected, the pulmonary valve underwent commissurotomy, and the hypoplastic pulmonary artery was augmented with a treated pericardial patch to accommodate increased postoperative flow. The tricuspid valve was tested and found competent, and the patient was weaned from bypass in stable sinus rhythm. The case-specific operative strategy is illustrated in Figures 1B–D. Her immediate postoperative course in the cardiac care unit was uneventful on minimal inotropic support, and postoperative chest radiography demonstrated a satisfactory cardiac silhouette and lung expansion. At two months' follow-up, her oxygen saturation had normalized to 98%, echocardiography showed no residual VSD or interatrial shunting, and the RVOT peak gradient had decreased to 73 mmHg. At six months postoperatively, repeat echocardiography showed continued clinical and echocardiographic improvement, with right ventricular diameter reduced from 4.38 cm to 3.98 cm, right atrial area reduced from 23.4 cm² to 17.9 cm², and complete resolution of cyanosis. The RVOT gradient decreased from 124 mmHg preoperatively to 73 mmHg at two months and 67 mmHg at six months; however, this represented persistent significant residual RVOT obstruction rather than a fully satisfactory hemodynamic result. Because CMR was not available at the time of the case, RV volumes and RV ejection fraction could not be quantified, and the echocardiographic changes were therefore interpreted as suggestive of favorable remodeling rather than definitive proof of RV reverse remodeling (Figures 3, 4).

## Discussion

This case represents an exceptionally rare survival of unrepaired TOF into the fifth decade of life and underscores the central role of multimodal cardiovascular imaging in the diagnosis and management of adult congenital heart disease. The delayed recognition of TOF in this patient highlights the diagnostic challenge of congenital heart disease presenting in older adults, particularly when survival into the fifth decade creates an atypical clinical context and prior documentation is limited. Although she had reportedly undergone echocardiographic evaluation before presentation to our hospital, the available outside records did not establish a diagnosis of TOF. This underscores the importance of maintaining clinical suspicion for ACHD when adults present with unexplained cyanosis, right-sided murmurs, or unexpectedly low oxygen saturation. The patient's ability to tolerate severe cyanotic congenital heart disease through 13 pregnancies and 11 live births is highly unusual. Pregnancy in women with ACHD requires lesion-specific risk stratification, ideally before conception and under the care of a multidisciplinary pregnancy heart team. In the modified WHO classification, unrepaired cyanotic congenital heart disease is generally considered high risk, while repaired TOF without major residual lesions is usually better tolerated. In uncorrected TOF, maternal risk is driven by fixed RVOT obstruction, limited ability to augment cardiac output, arrhythmia risk, thromboembolism, heart failure, and worsening hypoxemia, while fetal risk includes miscarriage, fetal growth restriction, prematurity, fetal loss, neonatal death, and recurrent congenital heart disease. These risks are particularly influenced by baseline maternal oxygen saturation, ventricular function, RVOT obstruction severity, and associated pulmonary vascular disease. Therefore, the patient's survival through 13 pregnancies without a recognized diagnosis is exceptional and highlights missed opportunities for earlier detection during antenatal and hospital-based care (4). Review of the available obstetric and medical records showed that oxygen saturation was not consistently documented during earlier encounters, despite routine recording of other vital signs. In chronically cyanotic adults who remain hemodynamically stable, hypoxemia may be less immediately apparent than in acute illness and may not prompt further investigation unless it is systematically measured, confirmed, and interpreted in clinical context. This pattern reflects a broader systems issue: clinicians, nurses, and other medical staff may be less likely to investigate unexplained hypoxemia in adults who are not acutely ill, particularly in resource-limited settings. In this context, cardiovascular imaging provided the key diagnostic and operative-planning information, with TTE and TEE defining the principal TOF anatomy, quantifying the severity of RVOT obstruction, assessing right and left ventricular systolic function, suggesting a possible PFO, and supporting the feasibility of a valve-sparing repair, while cardiac catheterization confirmed invasive hemodynamics and excluded significant coronary artery disease. In ACHD, CMR and cardiac CT provide important complementary information, particularly for RV volumes and function, pulmonary valve assessment, pulmonary artery anatomy, extracardiac vascular structures, and collateral vessels. In this case, CMR was not clinically available in the country at the time of the patient's evaluation and was therefore not performed; this service has since been introduced at our center, which became the first in the country to provide cardiac MRI. Cardiac CT was also not performed because the diagnostic anatomy was clearly demonstrated by TTE/TEE and contrast imaging, while catheterization and coronary angiography provided invasive hemodynamic and coronary assessment before surgery. However, we acknowledge that cross-sectional imaging would have strengthened preoperative anatomical characterization, particularly regarding RV volumes, branch pulmonary arteries, and possible aortopulmonary collateral vessels. Therefore, future similar adult presentations should ideally undergo CMR and/or cardiac CT when feasible as part of comprehensive ACHD evaluation (5, 6).

Surgical correction in adults with TOF differs from pediatric cases because long-standing RV pressure overload and fibrosis complicate RVOT muscle resection, and chronically hypoplastic pulmonary arteries often require augmentation; nevertheless, in this case, VSD patch closure, pulmonary valve commissurotomy, and pulmonary artery plasty were accomplished safely, avoiding the need for transannular patching or conduit placement and thereby preserving valvular function. Longitudinal echocardiographic follow-up demonstrated improvement in oxygen saturation, symptoms, right-sided chamber dimensions, and RVOT gradients, suggesting favorable postoperative remodeling after relief of chronic RV pressure overload. However, these findings should be interpreted cautiously. Echocardiography is useful for serial follow-up, but CMR is the preferred modality for quantitative assessment of RV volumes and function in TOF. Because CMR was not available at the time of this case, postoperative RV volumes and RV ejection fraction could not be measured. Therefore, the observed reduction in RV diameter and RA area should be considered supportive echocardiographic evidence of improvement rather than definitive proof of RV reverse remodeling. In addition, the residual RVOT gradient of 67 mmHg at six months remained significant, indicating residual obstruction and the need for continued ACHD surveillance (7).

From an imaging standpoint, this case emphasizes the value of integrating TTE, TEE, contrast studies, and invasive hemodynamics to define anatomy and physiology in adults with suspected congenital disease. Serial echocardiography provided a noninvasive means of tracking clinical and echocardiographic improvement, residual RVOT obstruction, and right-sided chamber dimensions after repair, illustrating how longitudinal imaging can guide long-term management in late-presenting TOF.

This case has several limitations. Preoperative CMR and cardiac CT were not performed; CMR was not clinically available in the country at the time, and CT was not pursued because echocardiographic, invasive, angiographic, and intraoperative findings were considered sufficient for operative planning. Consequently, RV volumes, branch pulmonary artery anatomy, and potential collateral vessels were not assessed by cross-sectional imaging. Pulmonary vascular resistance, wedge pressure, and Qp:Qs were also unavailable, limiting interpretation of the mildly elevated mean pulmonary artery pressure of 25 mmHg. Detailed obstetric and neonatal records were also incomplete, limiting assessment of pregnancy-specific outcomes such as preterm birth, delivery mode, birthweight, neonatal complications, and congenital heart disease among offspring.

## Conclusion

Overall, this case emphasizes that even in older adults, clinicians should remain vigilant for congenital heart disease as a potential cause of unexplained cyanosis. Detailed cardiovascular imaging can identify surgically remediable pathology, support an individualized valve-sparing surgical strategy, and demonstrate clinical improvement with echocardiographic findings suggestive of favorable right-sided remodeling. This case also highlights the importance of systematically interpreting low oxygen saturation values and maintaining expertise in adult congenital heart disease, especially in resource-limited settings.

## Data Availability

The data supporting the findings of this study will be made available by the corresponding author upon reasonable request.
